# Supplementary material for: Aurora Borealis (Bora), Which Promotes Plk1 Activation by Aurora A, Has an Oncogenic Role in Ovarian Cancer
Source: Cancers (Basel). 2020 Apr 6;12(4):886. doi: 10.3390/cancers12040886 (PMC7226261; doi:10.3390/cancers12040886)
Supplement: Supplementary file 1 [file cancers-12-00886-s001.pdf]

Supplementary Materials: *Aurora Borealis* (Bora), Which Promotes Plk1 Activation by Aurora A, Has an Oncogenic Role in Ovarian Cancer

Alfonso Parrilla, Marta Barber, Blanca Majem, Josep Castellví, Juan Morote, José Luis Sánchez, Asunción Pérez-Benavente, Miguel F. Segura, Antonio Gil-Moreno and Anna Santamaria

a

ENRICHED KEGG PATHWAYS			
TERM	Category	Genes	*p value*
KEGG_PATHWAY	Cell cycle	33	2,90E-06
KEGG_PATHWAY	Pathways in cancer	74	3,80E-06
KEGG_PATHWAY	Proteoglycans in cancer	42	5,70E-05
KEGG_PATHWAY	Ras signaling pathway	44	2,30E-04
KEGG_PATHWAY	Oocyte meiosis	26	3,60E-04
KEGG_PATHWAY	Fc gamma R-mediated phagocytosis	21	6,50E-04
KEGG_PATHWAY	p53 signaling pathway	18	7,50E-04
KEGG_PATHWAY	PI3K-Akt signaling pathway	58	1,10E-03
KEGG_PATHWAY	Circadian entrainment	22	1,30E-03
KEGG_PATHWAY	Dopaminergic synapse	27	1,50E-03
KEGG_PATHWAY	Small cell lung cancer	20	2,00E-03
KEGG_PATHWAY	Rap1 signaling pathway	38	2,60E-03
KEGG_PATHWAY	Glutamatergic synapse	24	2,90E-03
KEGG_PATHWAY	HIF-1 signaling pathway	21	3,60E-03
KEGG_PATHWAY	Axon guidance	25	5,80E-03

b

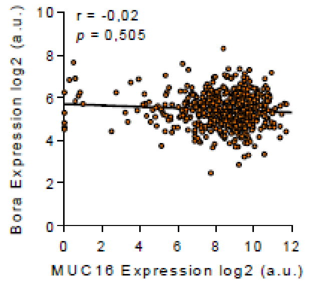

c

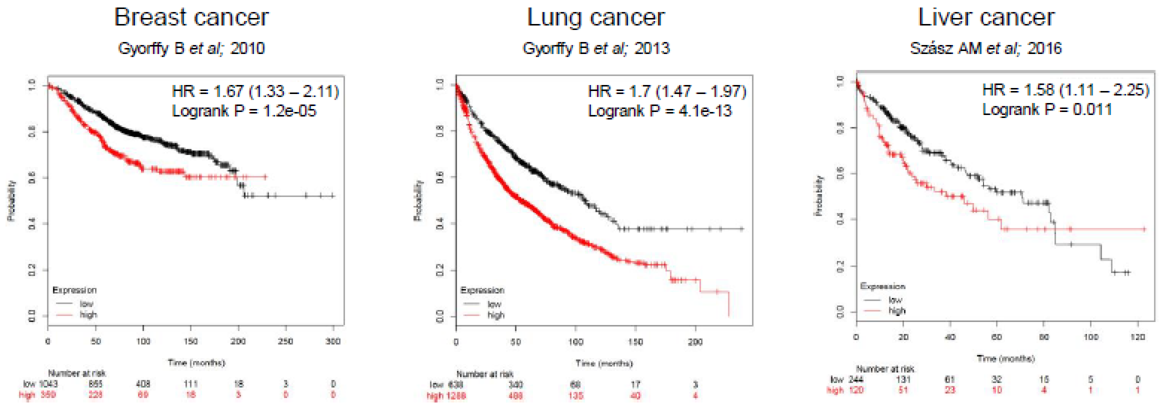

d

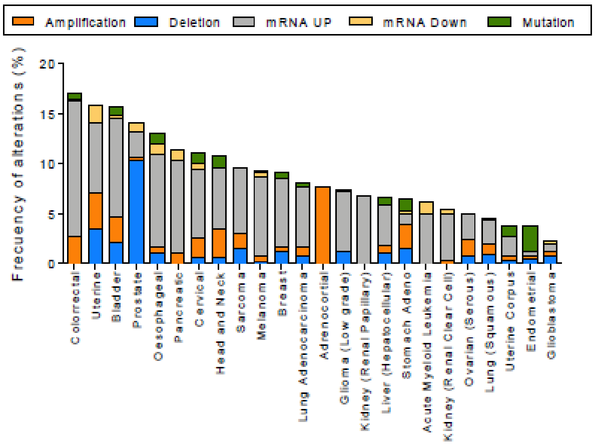

e

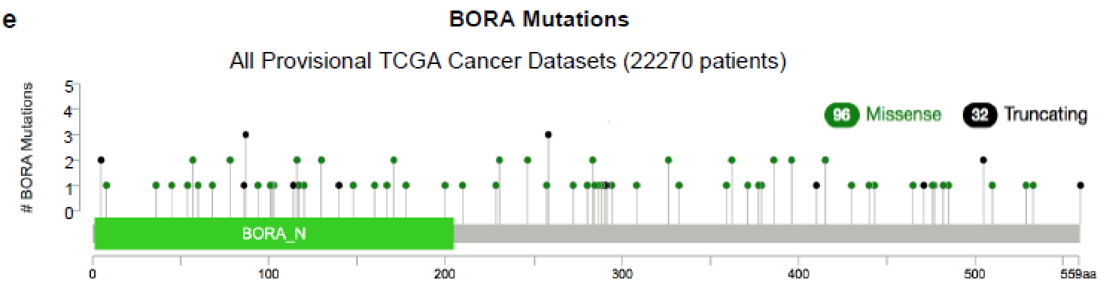

Figure S1. BORA expression is linked to poor prognosis (a) Functional.annotation of differentially expressed genes as reported by DAVID Bioinformatics 6.8. Enriched KEGG pathways using all differentially expressed genes were plotted. **(b)** Correlation between BORA and *MUC16* expression levels (CA-125 antigen) using the ovarian TCGA cohort. **(c)** Kaplan–Meier survival analysis based on the expression levels of BORA in breast, lung and liver carcinomas. *P*-values were estimated using a log-rank test to determine the difference in outcomes between patients with higher BORA expression

levels (red) *versus* those with lower/no levels (black). **(d)** Frequency (%) of BORA mutations and/or copy number alterations (deletions or amplifications) across the spectrum of human cancers currently annotated in the TCGA provisional **(e)** Histogram of BORA protein showing the mutational profile across the length of the protein. Data were retrieved from the TCGA databases using the cBioPortal website.

Cell Division	Mitotic process	Correlation with Survival: Worse if (p-value)	Ovarian cancer related function	
SPC25	+	High (0.0006)	+	
BORA	+	High (0.0216)	+	
CDCA5	+	High (0.0069)	+	
CCNA	+	High (0.0199)	+	
FAM64A	+	High (0.0137)	+	
KIF20B	+	High (0.0007)	+	
OPI5	+	High (0.0337)	+	
SPC24	+	High (1.1e-5)	+	
ARF6	-	Low (0.0259)	-	Broner et al., 2017
BUB1B	+	High (0.0007)	-	Sun et al., 2017
BUB1	+	High (0.0029)	-	Sun et al., 2017
CKS1B	-	High (0.0002)	-	Kawahara et al., 2017
CKS2	-	High (0.0046)	+	
CABLES1	-	High (0.0322)	-	Sakamoto et al., 2008
ERCC6L	-	High (0.2838)	+	
NEK2	+	High (0.0463)	-	Liu et al., 2014
NUF2	+	High (2.9e-5)	-	Sethi et al., 2012
ARHGEF2	+	Low (0.0501)	+	
SAC3D1	+	High (0.2219)	+	
TPX2	+	High (0.0013)	-	Tian et al., 2018
ZWINT	-	High (0.0021)	-	Xu et al., 2016
AURKA	+	High (9.6e-6)	-	Chiba et al., 2017
BIRC5	+	High (0.1289)	-	Wang et al., 2018
CDC20	+	High (0.0745)	-	Gayyed et al., 2016
CDC25A	+	High (0.0117)	-	Brogini et al., 2000
CDC25C	+	High (0.2129)	-	Gao et al., 2018
CDC6	+	High (0.1469)	-	Deng et al., 2016
CDC7	-	High (0.1324)	-	Kulkarn et al., 2009
CDCA3	+	High (4.5e-5)	-	Itzel et al., 2015
CDCA8	-	High (0.2284)	-	Wrzeszczynski et al., 2011
CENPE	-	High (0.0052)	-	Chong et al., 2018
CENPF	+	High (3.5e-5)	-	Xu et al., 2016
CCNB1	-	High (1.1e-9)	-	Ye et al., 2015
CCNB2	+	High (0.0488)	-	Fridley et al., 2018
CCNB3	-	High (0.0193)	+	
CCNE1	-	High (0.001)	-	Ayhan et al., 2017
CCNE2	-	High (0.0005)	-	Xie et al., 2017
CCNY	-	High (0.1277)	-	Liu et al., 2016
CDK1	+	High (0.0006)	-	Yang et al., 2016
FAM83D	+	High (8.1e-6)	-	Ramakrishna et al., 2010
HMGA2	+	High (0.0364)	-	Wu et al., 2011
KIF11	+	High (0.0016)	-	Xu et al., 2016
KIF14	-	High (2.5e-5)	-	Qiu et al., 2017
KIF18B	-	High (0.0132)	-	Itzel et al., 2015
KIF2C	+	High (0.0377)	-	Zhao et al., 2014
KIFC1	-	High (0.0104)	-	Mittal et al., 2016
NCAPG	-	High (0.0022)	+	
HNCAPH	-	High (0.0172)	+	
PTTG1	-	High (0.0498)	-	Nakachi et al., 2016
PSRC1	-	High (0.0221)	+	
RCC2	-	High (1.4e-5)	-	Wu et al., 2018
SETP11	-	High (0.0433)	+	
SPAG5	-	High (0.0009)	+	
SMC1A	-	High (0.0443)	-	Liu et al., 2014
TIMELESS	+	Low (0.0165)	-	Jim et al., 2015

Figure S2. Integrative computational analysis reveals druggable.mitotic proteins to explore in OC. (a) Genes listed according to the different filters. The "+" and "-" symbols refer to (1) included or not in the mitotic process GO term or (2) if the gene or protein –function in OC is reported or not in the literature. High and low refers to the gene expression correlated with worse survival outcome. Survival analysis were carried out using the Kaplan Meier Plotter platform. References for those genes analyzed.

in the literature are indicated.

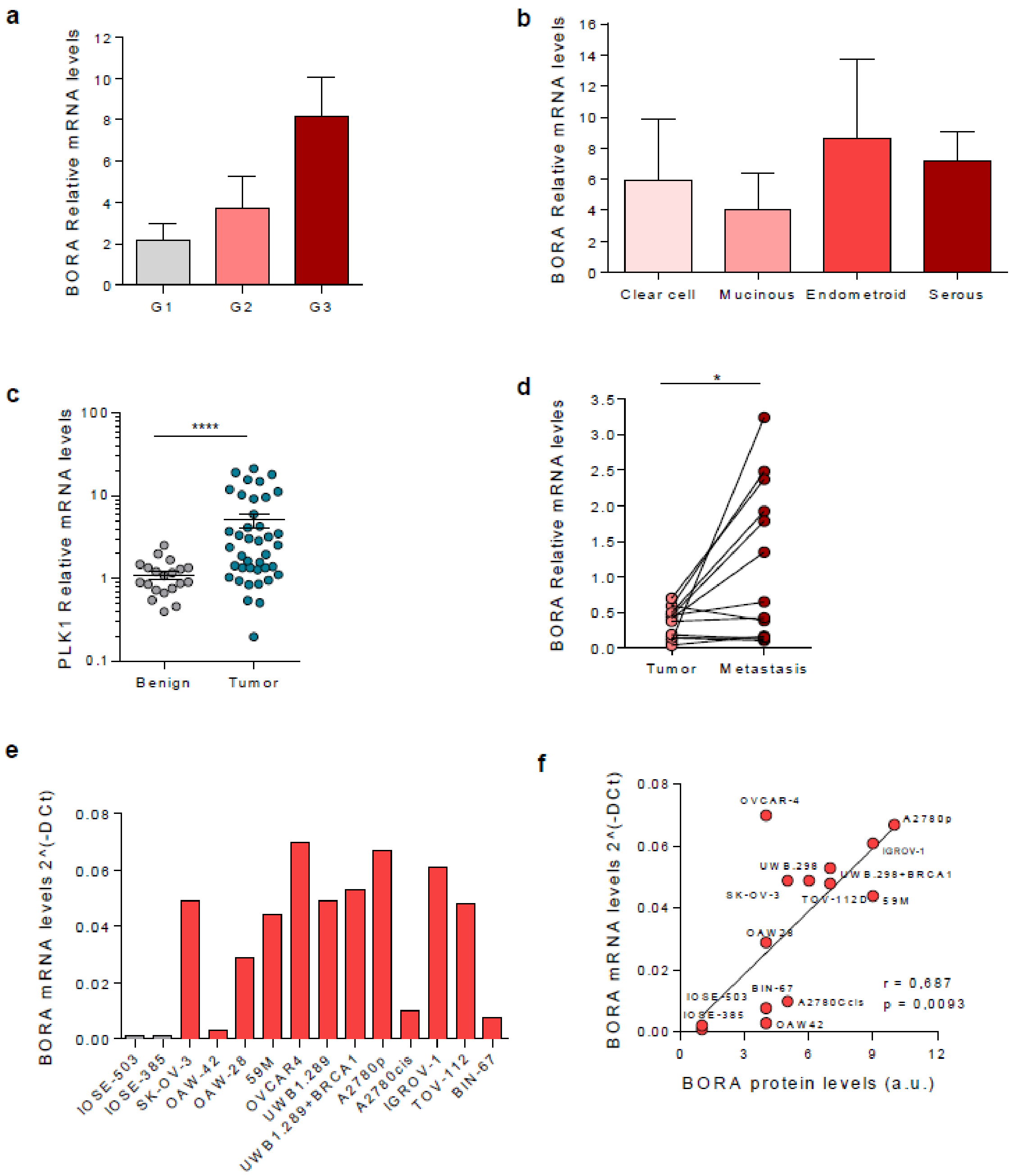

Figure S3. BORA expression in human samples and ovarian cell lines. **(a-b)** BORA relative mRNA levels from tumor samples (n=40) categorized by the neoplasm grade and the histological OC subtypes. **(c)** PLK1 mRNA expression levels in the collection of ovarian samples **(d)** Graph represents BORA relative expression of the primary ovarian tumoral tissue to its paired metastatic sample. MRNA expression levels of each sample were normalized to its respective levels of *GAPDH* expression. The relative fold-change in expression was determined by the comparative 2(-ΔΔCt)

method and normalized against *BORA* expression value from the primary tumor. **(f)** MRNA levels of *BORA* in the spectrum of ovarian cell lines. **(g)** Correlation (Spearman) between *BORA* mRNA and protein levels in the ovarian cell lines. In (c) and (e), *P*-values were calculated using unpaired Student's *t*-test. ***p<0,001.

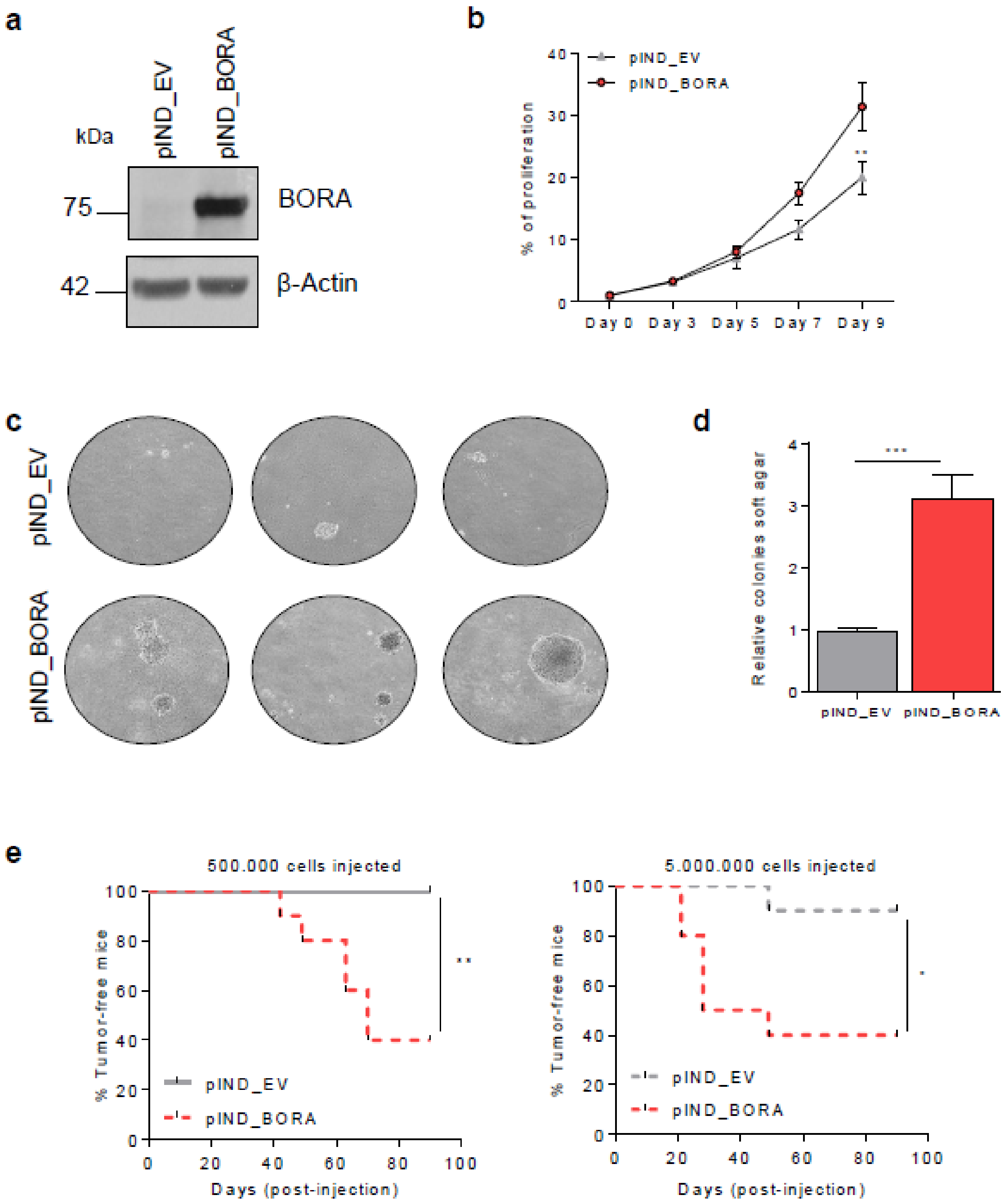

Figure 4. BORA overexpression enhances the tumoral aggressiveness status in the SK-OV-3 cell line. (a) Immunoblot showing BORA overexpression in the EV- and BORA- SK-OV-3-transduced cells upon doxycycline administration (0,25 µg/mL). β-Actin was used as loading control. **(b-d)** Average quantification of proliferation and capacity to growth in soft agar conditions. Graph represent mean ± SEM of at least three independent experiments. *P*-values were calculated using unpaired Student's *t*-test. **P<0,01; ***P<0,001. **(e)** Diffuse tissue engraftment appearance in the flank of the mice depicted in a graph after subcutaneous injection of pIND_EV- and pIND_BORA- IOSE transduced cells into the flank of the mice. Two approaches were followed: one injecting 5·105 cells and other with 5·106 cells. *p*-values were estimated using a log-rank test to determine the difference in appearance between pIND_EV tumors (grey line) *vs* pIND_BORA tumors (red line). *p<0,05; **p<0,01.

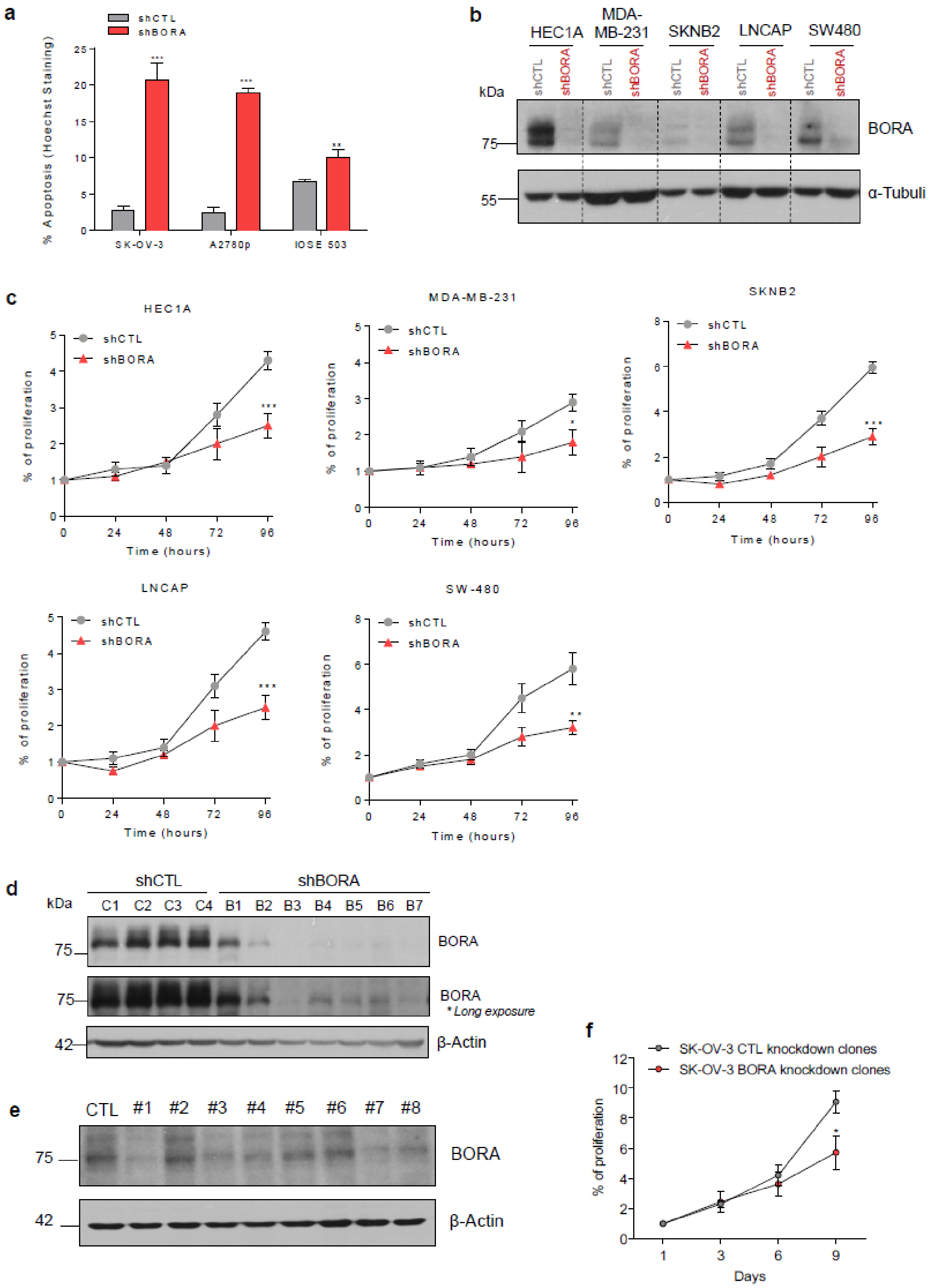

Figure 5. BORA is essential to OC viability. (a) Average quantification of cell death assays in SK-OV-3, A2780p and IOSE cells at 96h post lentiviral transduction. Graphs represent mean ± SEM of three independent experiments **(b)** Representative immunoblot of BORA knockdown in endometrial, breast, neuroblastoma, prostate and colon carcinoma cell lines. α-Tubulin was used as loading control. **(c)** Normalized proliferation curve of shCTL (grey line) and

shBORA (red line) -transduced cells in the different tumor cell lines. **(d)** Immunoblot analysis of BORA in control- and BORA- depleted A2780p clones. **(e)** Immunoblot of different SK-OV-3 CRISPR/cas9 clones and **(f)** proliferative curves of some of these clones. β-Actin was used as loading control. Graphs represent mean ± SEM of three independent experiments. In C and F, *P*-values were calculated using unpaired Student's *t*-test. *$p < 0,05$; **$p < 0,01$; ***$p < 0,001$.

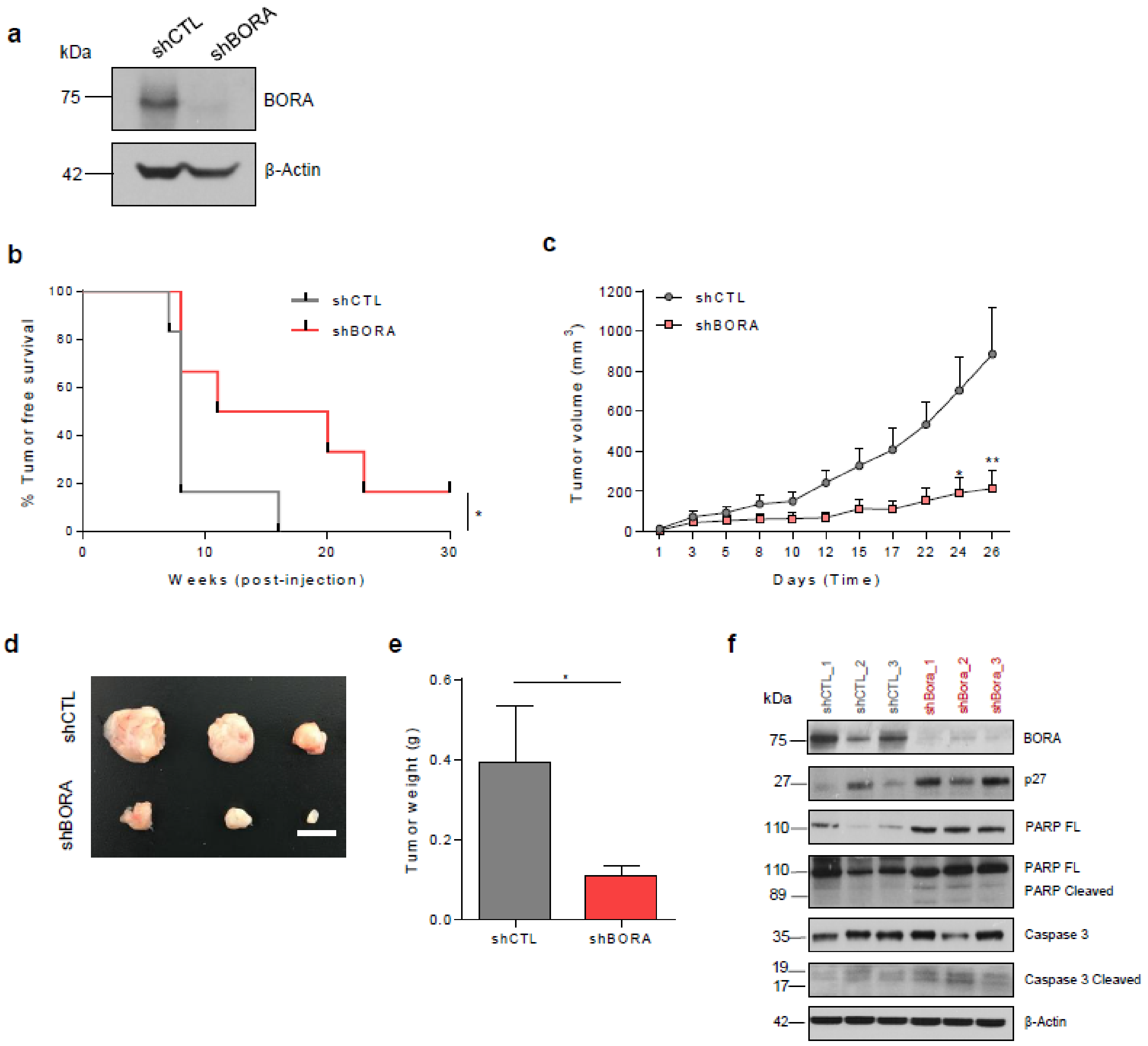

Figure S6. BORA impacts on tumor engraftment. (a) A portion of shCTL and shBORA transduced cells used for the *in vivo* model were analyzed by immunoblot showing BORA downregulation. β-Actin was used as loading control. (b) Tumor engraftment incidence. *P*-value was estimated using a log-rank test to determine the difference in appearance between shCTL tumors (grey line) *vs* shBORA tumors (red line). *P<0,05**.** **(c)** Tumor volume was monitored over time using electronic caliper. Two-way ANOVA was used to calculate the significance of the difference between shCTL (grey line) and shBORA tumors (red line). *P<0,05; **P<0,01. **(d)** Macroscopic images of resected tumors at end-point. Bar: 1 cm. **(e)** Average weight of the tumors taken at the time of the resection. *P*-value was calculated using a two-tailed Student's *t*-test. *P<0,05. **(f)** Immunoblot analysis of BORA, p27, PARP and Caspase 3 protein markers using protein lysates from representative xenografts from both experimental groups. β-Actin.

was used as loading control.

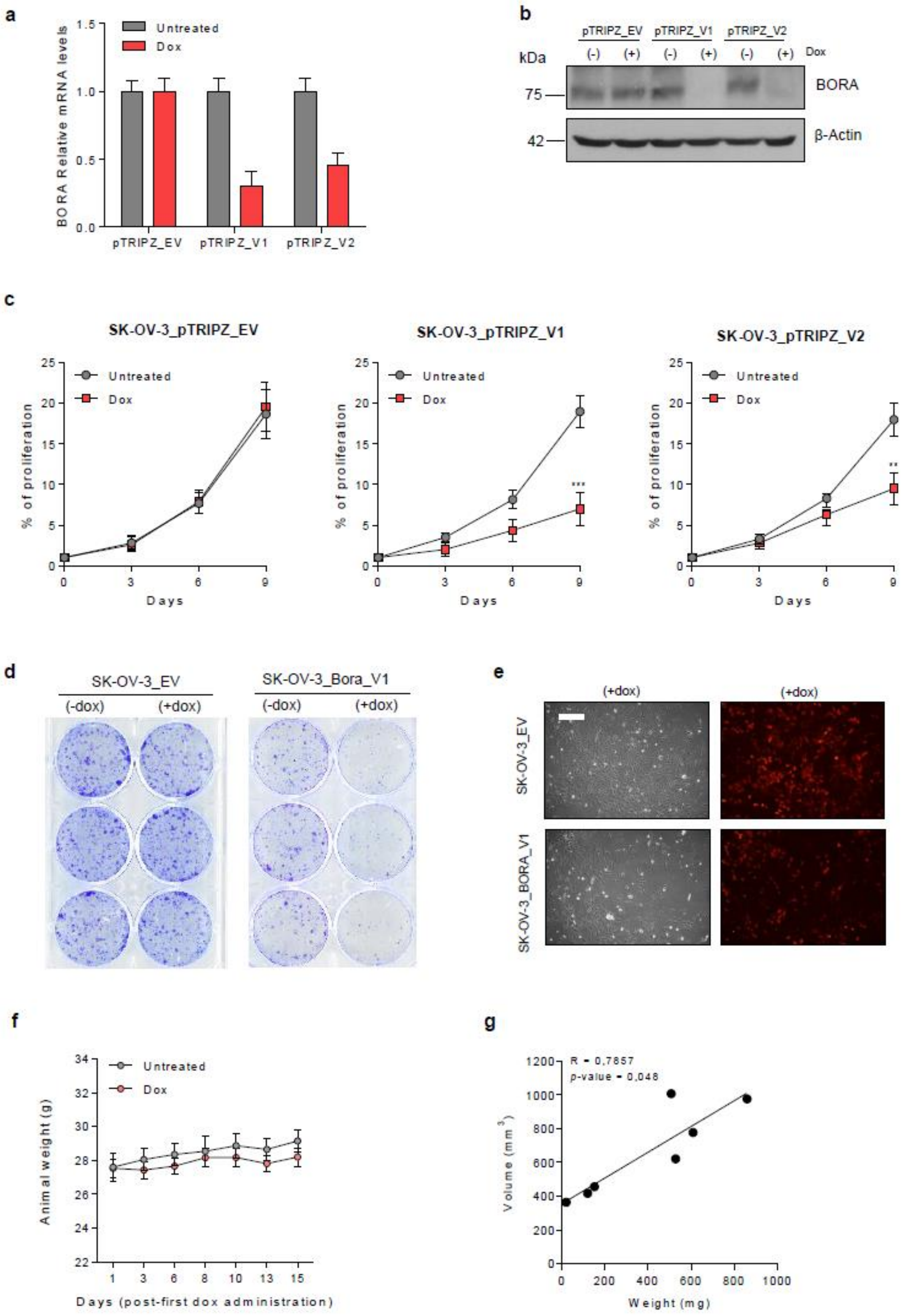

Figure S7. BORA depletion using an inducible system impairs proliferation and colony formation capacities. (a) Relative expression of BORA levels analyzed by RT-qPCR in the different stable pTRIPZ transduced SK-OV-3 cells upon doxycycline administration (1 μg/mL). *GAPDH* was used as endogenous control. The relative fold-change in expression was determined by the comparative 2(-

ΔΔCt) method and normalized against control (untreated) expression value. **(b)** Immunoblot showing effective BORA inducible depletion upon doxycycline treatment. **(c)** Proliferation time course comparing pTRIPZ_EV or pTRIPZ_BORAV1 or pTRIPZ_BORAV2 treated or untreated with 1 μg/mL of doxycycline. *P*-value was calculated using a two-tailed Student's *t*-test. ***P*<0,01; ****P*<0,001. **(d)** Representative images of a colony formation assay with pTRIPZ_EV and pTRIPZ_BORAV1 transduced cells treated with or without doxycycline and allowed to grow for 10-12 days. **(e)** Representative images of pTRIPZ- EV and pTRIPZ_BORA V1– SK-O-3 infected cells. Expression of the pTRIPZ vector is followed by the expression of TurboRFP protein. **(f)** Spearman correlation between volume and weight of shBORA-depleted tumors. **(g)** Animal weight of untreated and doxycycline treated-animals during the consecution of the experiment, indicating a good doxycycline tolerability in treated-mice. Graphs represent mean ± SEM of three independent experiments.

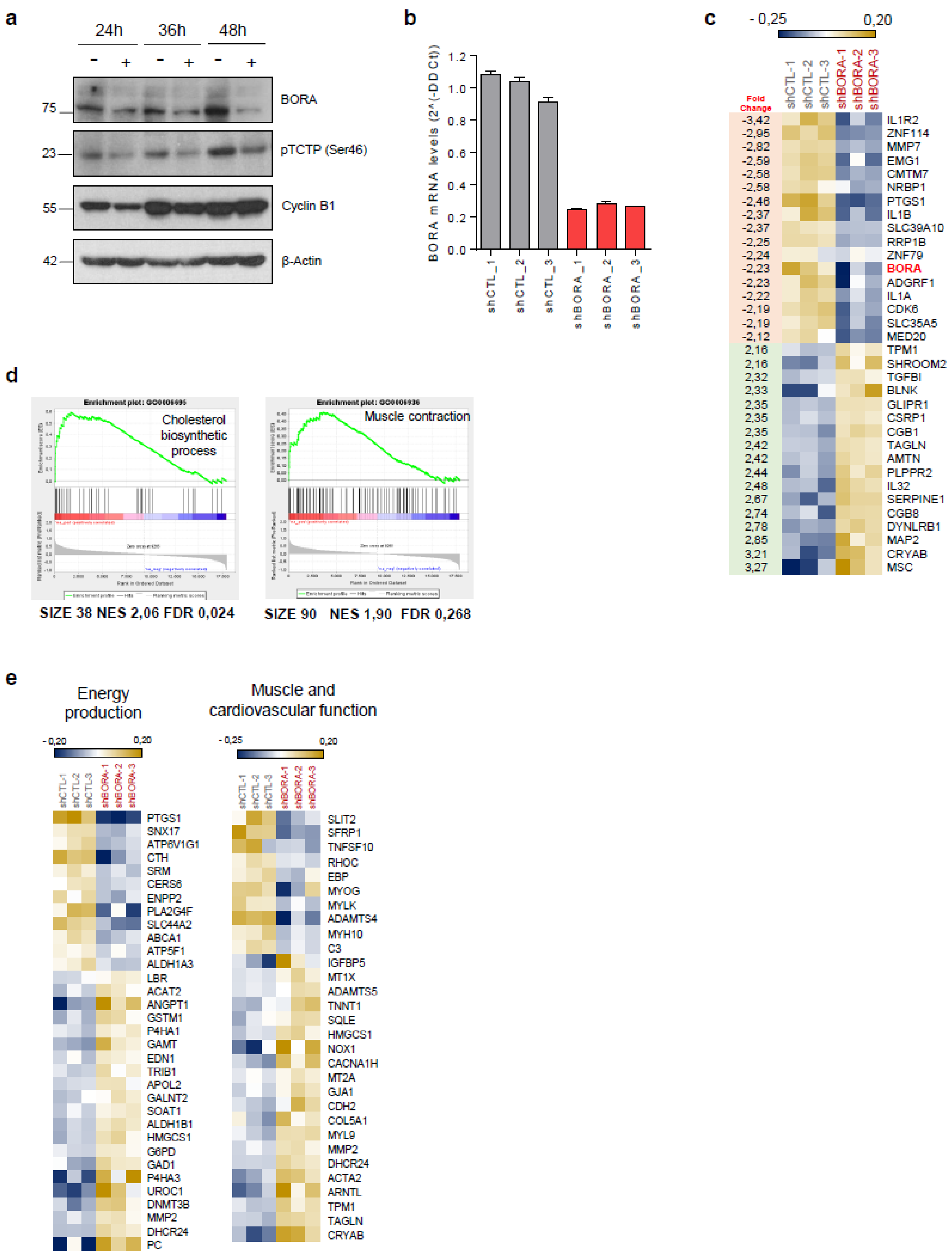

Figure S8. BORA alters the expression of genes involved in energy production and muscle and cardiovascular processes. (a) Time course immunoblot of BORA, Cyclin B1 and pTCTP (Ser46) to select the best time to deplete BORA and see the causes of the depletion more than the consequences. β-Actin used as loading control. **(b)** BORA mRNA levels performed in the samples used to the microarray analysis. *GAPDH* was used as endogenous control. **(c)** Representative genes with the highest fold change variation upon BORA depletion. **(d-e)** Enrichment plots and heat maps showing the transcriptomic impact of BORA silencing in genes involved in energy production and muscle and cardiovascular functions. The color key shows relative expression levels of the differentially expressed

genes (yellow corresponds to overexpressed genes while blue corresponds to underexpressed genes).

98

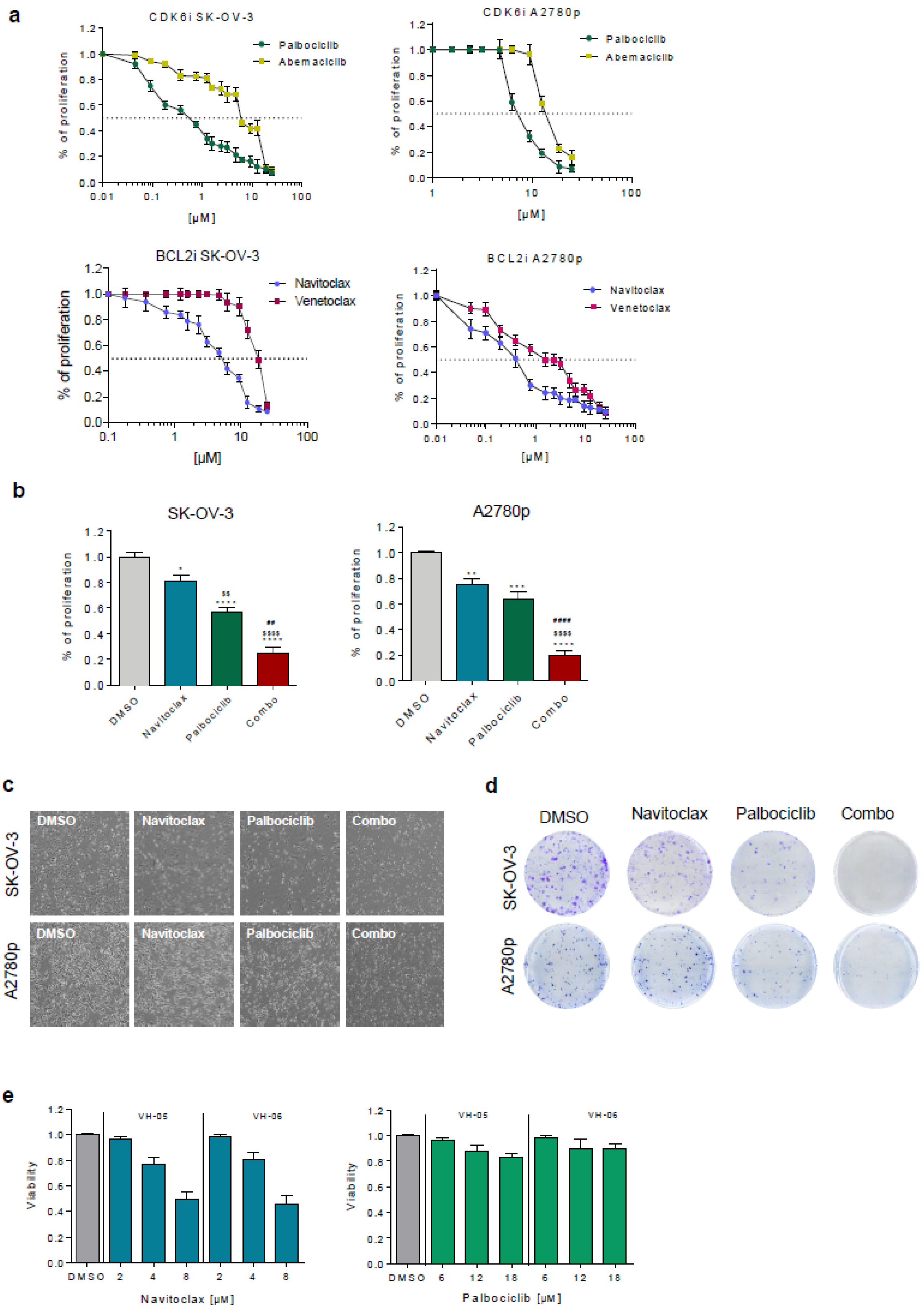

Figure S9. BCL-2 and CDK6 inhibitors reduce the proliferative capacity of OC cells. (a) Normalized proliferation curves of the indicated OC cell lines treated with CDK6 inhibitors: Palbociclib and Abemaciclib and BCL-2 inhibitors: Venetoclax and Navitoclax for 5 days. Drug doses ranges from

0,01 μM to 25 μM. Data represent an average quantification of three independent experiments ± SEM (n=6/condition). **(b)** Proliferation assay of the indicated cell lines treated with the two agents at the best CI for five days, measured by crystal violet staining (n=6/condition). Graphs are the average of three independent experiment ± SEM. *P*-value was calculated using One-way ANOVA. * compares DMSO *versus* the rest of the conditions; # *Navitoclax versus* rest of conditions; $ *Palbociclib versus* Combo. *,#,$*P*<0,05; **,##,$$*P*<0,01; ***,###,$$$*P*<0,01; ****,####,$$$$*P*<0,001 **(c)** Representative macroscopic images of SK-OV-3 and A2780p cells lines treated with the inhibitors as in (b). Bar: 100 μm **(d)** Colony formation capacity of the indicated cells lines treated with the two compounds alone and in combination. **(e)** MTS assay with different suboptimal drug concentrations of Palbociclib and Navitoclax tested in the two patient-derived tumoral cells grown in 3D

Table S1. Data sets, bioinformatic tools and techniques used in this study, with the corresponding references.

Dataset	Number of samples	Type	Status	References	Tool
GSE14407	24	OC	Public	-	GEO2R
GSE26712	195	OC	Public	-	GEO2R
GSE27651	41	OC	Public	-	GEO2R
GSE38666	30	OC	Public	-	GEO2R
GSE54388	22	OC	Public	-	GEO2R
Kaplan-Meier Plotter	1656	OC	Public	Gyorffy B *et al.,* (2012)	http://kmplot.com
Kaplan-Meier Plotter	1402	Breast	Public	Gyorffy B *et al.,* (2010)	http://kmplot.com
Kaplan-Meier Plotter	1926	Lung	Public	Gyorffy B *et al.,* (2013)	http://kmplot.com
Kaplan-Meier Plotter	364	Liver	Public	Menyhart O *et al.,* (2018)	http://kmplot.com
cBioPortal	-	23 types	Public	Gao *et al.,* (2013)	http://www.cbioportal.org/
DAVID	-	-	Public	Huang W *et al.,* (2007)	http://david.abcc.ncifcrf.gov
GSEA	-	-	Public	Subramanian *et al.,* (2005)	http://www.broad.mit.edu/gsea/
Venny diagram	-	-	Public	Oliveros, J.C. (2007-2015)	http://bioinfogp.cnb.csic.es/tools/venny
R2 genomics	-	-	Public	-	http://r2.amc.nl

Supplementary References

Gyorffy B, Lanczky A, Szallasi Z. Implementing an online tool for genome-wide validation of survival-associated biomarkers in ovarian-cancer using microarray data of 1287 patients, Endocrine-Related Cancer. 2012 Apr 10;19(2):197-208

Table S2. Fresh-frozen tissue samples of the ovary for mRNA analysis.

N	Group	Type	FIGO stage	Grade
1	B	Folicular cyst	-	-
2	B	Folicular cyst	-	-
3	B	Folicular cyst	-	-
4	B	Folicular cyst	-	-
5	B	Folicular cyst	-	-
6	B	Simple mucinous cyst.	-	-
7	B	Simple mucinous cyst.	-	-
8	B	Simple mucinous cyst.	-	-
9	B	Simple mucinous cyst.	-	-
10	B	Simple mucinous cyst.	-	-
11	B	Simple serous cyst.	-	-
12	B	Simple serous cyst.	-	-
13	B	Simple serous cyst.	-	-
14	B	Simple serous cyst.	-	-
15	B	Simple serous cyst.	-	-
16	B	Simple serous cyst.	-	-
17	B	Simple serous cyst.	-	-
18	B	Fibroma	-	-
19	B	Fibroma	-	-
20	B	Fibroma	-	-
21	Early	Mucinous	IC	2
22	Early	Mucinous	IIB	2
23	Early	Mucinous	IA	2
24	Early	Mucinous	IC	2
25	Early	Endometrioid	IA	3
26	Early	Endometrioid	IC	2
27	Early	Endometrioid	IC	2
28	Early	Endometrioid	IC	1
29	Early	Endometrioid	IC2	1
30	Early	Endometrioid	IA	3
31	Early	Clear cell	IIB	3
32	Early	Clear cell	IC	3
33	Early	Clear cell	IIB	3
34	Early	Papillary serous	IC	3
35	Early	Papillary serous	IIA	3
36	Early	Papillary serous	IIb	1
37	Early	Papillary serous	IA	3
38	Early	Papillary serous	IC1	3
39	Late	Clear cell	IIIC	3
40	Late	Clear cell	IIIC	3
41	Late	Not typified	IIIC	2
42	Late	Mucinous	IA	-
43	Late	Papillary serous	IIC	3
44	Late	Papillary serous	IIIC	3
45	Late	Papillary serous	IV	3
46	Late	Papillary serous	IV	3
47	Late	Papillary serous	IIIA	3
48	Late	Papillary serous	IIIC	3
49	Late	Papillary serous	-	3
50	Late	Papillary serous	IIIC	3
51	Late	Papillary serous	IIIC	3
52	Late	Papillary serous	IIIC	3
53	Late	Papillary serous	IIIC	3
54	Late	Papillary serous	IIIC	3
55	Late	Papillary serous	IV	3
56	Late	Papillary serous	IIIC	3
57	Late	Papillary serous	IIIC	3
58	Late	Papillary serous	-	3
59	Late	Papillary serous	IIIC	3
60	Late	Papillary serous	IIIC	

*B: benign ovary; Early and Late: stage of the primary tumors; “cyst.” means cystadenoma, a type of benign cyst of the ovary; “Grade” means grade of cell differentiation.

Table S3. Fresh-frozen tissue samples of the ovary for protein analysis.

N	Group	Type	FIGO stage	Grade
1	B	Simple serous cyst.	-	-
2	B	Simple serous cyst.	-	-
3	B	Simple serous cyst.	-	-
4	B	Fibroma	-	-
5	B	Simple serous cyst.	-	-
6	B	Fibroma		
7	T	Papillary serous	IV	3
8	T	Papillary serous	IV	3
9	T	Papillary serous	IIIC	3
10	T	Papillary serous	IVB	3
11	T	Papillary serous	IIIC	3
12	T	Papillary serous	IIIA1	3
13	T	Papillary serous	IIIB	3
14	T	Papillary serous	IIIB	3

*B: benign ovary; *T: Primary tumor; "Cyst." means cystadenoma, a type of benign cyst of the ovary; "Grade" means grade of cell differentiation.

Table S4. FFPE paired tumor and metastases

Patient	Type	FIGO	Grade	**Tumor** (Yes/No)	**Metastasis** (Yes/No)
1	Papillary serous	IIIC	3	Yes	Yes
2	Papillary serous	IIC	3	Yes	Yes
3	Papillary serous	IIIC	3	Yes	Yes
4	Papillary serous	IIIC	3	Yes	Yes
5	Papillary serous	IIIC	3	Yes	Yes
6	Papillary serous	IIIC	3	Yes	Yes
7	Papillary serous	IIIC	3	Yes	Yes
8	Papillary serous	IIIC	3	Yes	Yes
9	Papillary serous	IIIC	3	Yes	Yes
10	Papillary serous	NA	NA	Yes	Yes
11	Papillary serous	IIIC	3	Yes	Yes
12	Papillary serous	IIIC	3	Yes	Yes
13	Papillary serous	IV	3	Yes	Yes

NA: not available

Table S5. Patient-derived ascites from advanced stage OC

# (Patient)	Type	FIGO	Grade	Culture conditions
VH-01	Clear cell	IIIC	3	Mix medium: mixture (1:1) of MCDB 105 and M-199 mediums (Biological Industries, Israel), with 15% FBS, 2 mM L-glutamine, 100 U/mL penicillin and 100 μg/mL streptomycin (Invitrogen, CA, USA)
VH-02	Papillary serous	IIIC	3	
VH-03	Papillary serous	IIIA1	3	
VH-04	Papillary serous	IIIC	3	
VH-05	Papillary serous	IIIC	3	
VH-06	Papillary serous	IIIC	3	

*VH means Vall Hebron Hospital

Table S6. General characteristics of the used human ovarian cell lines

Ovarian Cancer Cell Line	Tumor Type	Source	Growth properties	Medium
**TOV112**	High-grade Endometrioid Adenocarcinoma	Primary tumor	Monolayer. *Morphology: epithelial*	Mixt medium: mixture (1:1) of MCDB 105 and M-199 mediums (Biological Industries, Israel)
**SKOV3**	Epithelial Ovarian Adenocarcinoma	Ascites	Monolayer. *Morphology: mesenchima*l	McCoy's 5A (Biowest)
**OAW42**	Epithelial Ovarian Adenocarcinoma	Ascites	Monolayer. *Morphology: mesenchima*l	DMEM High glucose (Biowest)
**OAW28**	High Grade Serous Carcinoma	Ascites	Monolayer. *Morphology: epithelial*	DMEM High glucose (Biowest)
**59M**	Endometrioid carcinoma of ovary (with clear cell components)	Ascites	Monolayer. Morphology: mesenchimal	DMEM High glucose (Biowest)
**OVCAR4**	High Grade Serous Carcinoma	Primary tumor	Monolayer. *Morphology: epithelial*	Mixt medium: mixture (1:1) of MCDB 105 and M-199 mediums (Biological Industries, Israel)
**A2780p**	High-grade Endometrioid Adenocarcinoma	Primary tumor	Monolayer. Morphology: *epithelial*	RPMI (Biowest)
**A2780cis***	High-grade Endometrioid Adenocarcinoma	Primary tumor	Monolayer. Morphology: *epithelial*	RPMI (Biowest)
**BIN-67**	Small cell carcinoma of the ovary hypercalcemic type (SCCOHT)	Primary tumor	Monolayer. Morphology: *epithelial*	100 mL de DMEM F12 (Biowest) + 100 mL DMEM High Glucose (Biowest) + 50 mL de FBS
**IGROV-1**	High-grade Endometrioid Adenocarcinoma	Primary tumor	Monolayer. Morphology: *epithelial*	RPMI (Biowest)
**IOSE 503**	Immortalized Ovarian Surface Epithelium	Ovarian surface tissue	Monolayer. *Morphology: epithelial*	Mixt medium: mixture (1:1) of MCDB 105 and M-199 mediums (Biological Industries, Israel)
**IOSE 385**	Immortalized Ovarian Surface Epithelium	Ovarian surface tissue	Monolayer. *Morphology: epithelial*	Mixt medium: mixture (1:1) of MCDB 105 and M-199 mediums (Biological Industries, Israel)
**UWB1.289/BRCA1MUT**	High Grade Serous Carcinoma	Primary tumor	Monolayer. Morphology: *mesenchymal*	1 : 1 mixture of medium RPMI (Biowest)+ MEGM (FBS 3%)
**UWB1.289 + BRCA1**	High Grade Serous Carcinoma	Primary tumor	Monolayer. Morphology: *mesenchymal*	1 : 1 mixture of medium RPMI (Biowest) + MEGM (FBS 3%) + G418

Footnote: *Resistant OC cell line to cisplatin, derived from the parental A2780

Table S7. List of antibodies used for Immunloblot and Immunohistochemistry

Antibody	Cataog number	Source	Application	Conditions
Aurora A	610938	BD Biosciencies	IB	1:1000 dilution, 5% nonfat milk
Bcl-2	M0887	DAKO	IB	1:1000 dilution, 5% nonfat milk
Bora	#12109	Cell Signaling	IB	1:1000 dilution, 5% nonfat milk
Caspase 3	#9665	Cell Signaling	IB	1:1000 dilution, 5% BSA
Caspase 3 Cleaved	#9661	Cell Signaling	IB	1:750 dilution, 5% BSA
Cdk6	#13331	Cell Signaling	IB	1:1000 dilution, 5% nonfat milk
Cyclin B1	#05-373	Merk Millipore	IB	1:1000 dilution, 5% nonfat milk
JNK1	#3708	Cell Signaling	IB	1:1000 dilution, 5% nonfat milk
Ki67	790-4286	Roche (Ventana Med.Syst.)	IHQ	-
mCherry	96752FR	Novus Biologicals	IB	1:1000 dilution, 5% nonfat milk
PARP1	#9542	Cell Signaling	IB	1:3000 dilution, 5% BSA
Plk1	#4535	Cell Signaling	IB	1:1000 dilution, 5% nonfat milk
pTCTP (Ser46)	#5251	Cell Signaling	IB	1:3000 dilution, 5% BSA
p27 Kip1 (D69C12)	#3686	Cell Signaling	IB	1:1000 dilution, 5% nonfat milk
p53	sc-126	Santa Cruz Biotechnology	IB	1:1000 dilution, 5% nonfat milk
p65	#8242	Cell Signaling	IB	1:1000 dilution, 5% nonfat milk
α-Tubulin	T9026	Sigma Aldrich	IB	1:5000 dilution, 5% nonfat milk
β-Actin	sc-47778	Santa Cruz Biotechnology	IB	1:10.000 dilution, 5% nonfat milk
anti-Rabbit IgG	A0545	Sigma Aldrich	IB	1:5000 dilution, 5% nonfat milk

anti-Mouse IgG	A9044	Sigma Aldrich	IB	1:5000 dilution, 5% nonfat milk; 1:10000 for α-Tubulin

"IB" means Immunoblot; "IHQ" means Immunohistochemistry

Figure 1L

Table S8. Primer sequences for genes detected by Sybr-Green RTqPCR technology

Gene Name	NM_number (GeneCards)	Catalog #	Primer sequence (5'-3')	Amplicon length
TPM1	NM_001018004	4689011001	ctctgaggctctcaaagatgc<br>cagctggatgcgtctgttc	104 nt
SHROOM2	NM_001649.3	4685016001	gaggtcccggtcttcacc<br>ctgccttcgcagttcgac	67 nt
MMP7	NM_002423.4	4685032001	cggatggtagcagtctaggg<br>aggttggatacatcactgcattag	111 nt
CDK6	NM_001145306.1	4684982001	tgatcaactaggaaaaatcttggac<br>ggcaacatctctaggccagt	70 nt
BCL2	NM_000633.2	4688988001	agtacctgaaccggcacct<br>gccgtacagttccacaaagg	74 nt
MAD2L1	NM_002358.3	4687655001	cgcgtgcttttgtttgtgt<br>gctgttgatgccgaatgag	117 nt
SFRP1	NM_003012.4	4687990001	gctggagcacgagaccat<br>tggcagttcttgttgagca	75 nt
CLASP2	NM_001207044.1	4689089001	cgaccaagtgtgagtcaagg<br>gatctggaatggtgtctggag	110 nt
MARK2	NM_017490.3	4685008001	tggaagtcgctggtagtcct<br>ccccgaatcatgttggac	95 nt
SLC25A10	NM_001270888.1	4688031001	cccgcagacttggtcaac<br>tacgcggtacaggccatc	99 nt
IL1B	NM_000576.2	4689011001	tacctgtcctgcgtgttgaa<br>tctttgggtaatttttgggatct	76 nt
RHOB	NM_004040.3	4688589001	gcatgaacaggacttgacca<br>ctgtgtcctccccaagtcag	71 nt
RERG	NM_032918.2	4689038001	aacttgcagaggaccgtagc<br>ttggaagagtccacaatcctg	64 nt
GAPDH	NM_002046	04689003001	caacgaccactttgtcaagc<br>ggtggtccaggggtcttact	115nt

The whole western blot images

Figure 1L

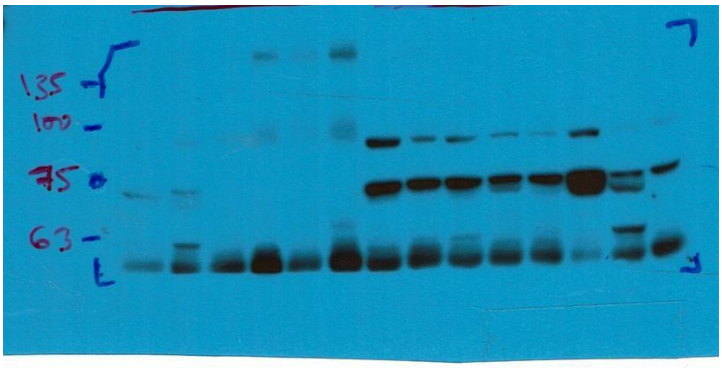

BORA

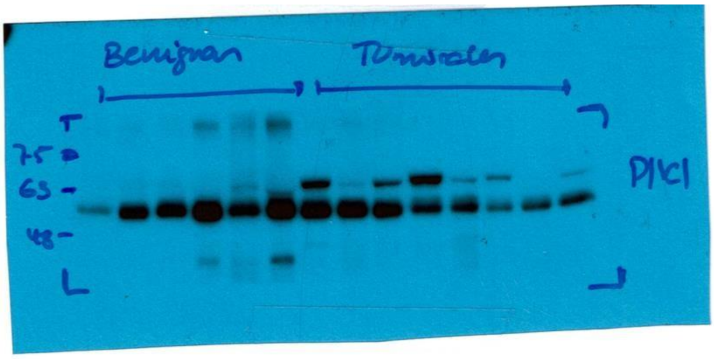

PLK1

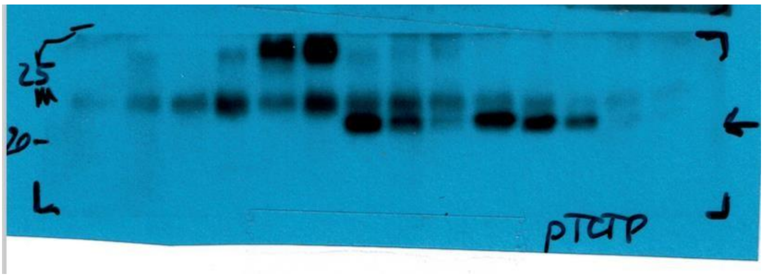

pTCTP (Ser46)

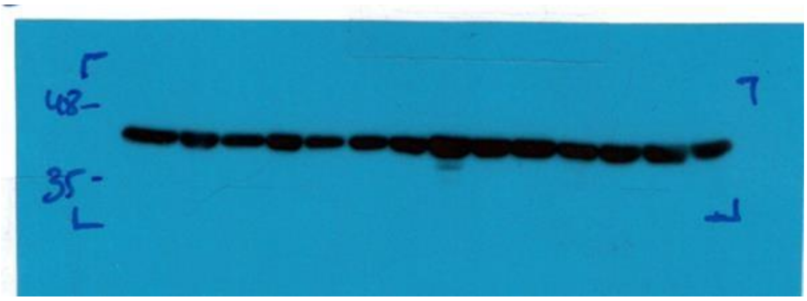

B-Actin

Figure 1M

Figure 1M

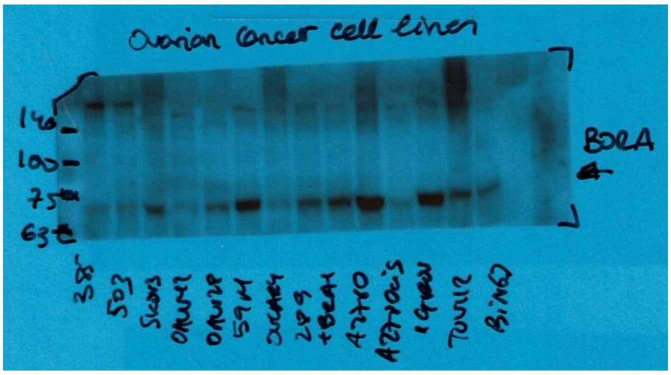

BORA

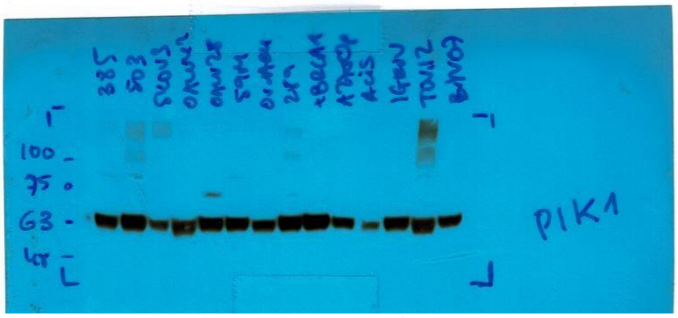

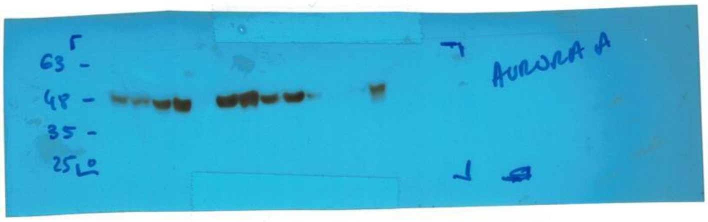

Plk1

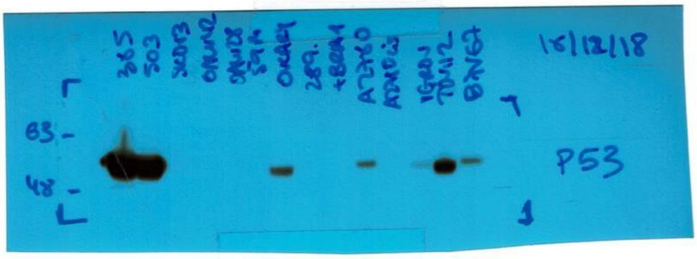

Aurora A

Figure 1M

p53

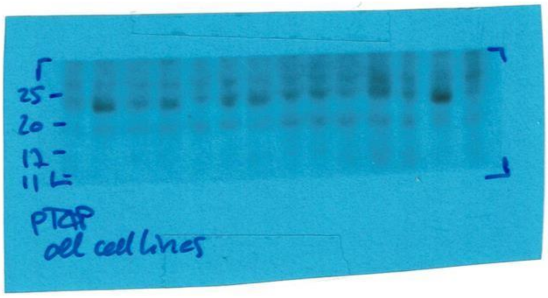

pTCTP (Ser46)

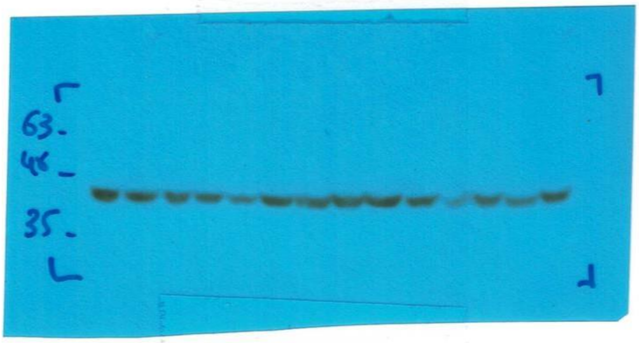

B-Actin

Figure 2A

Figure 2A

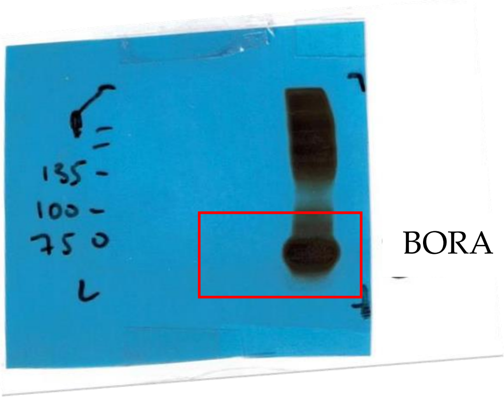

BORA

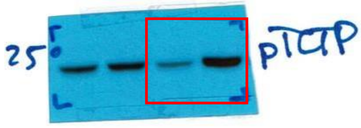

pTCTP (Ser46)

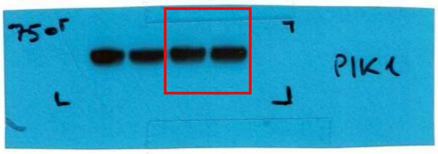

Plk1

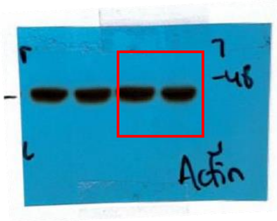

B-Actin

Figure 2H

Figure 2H

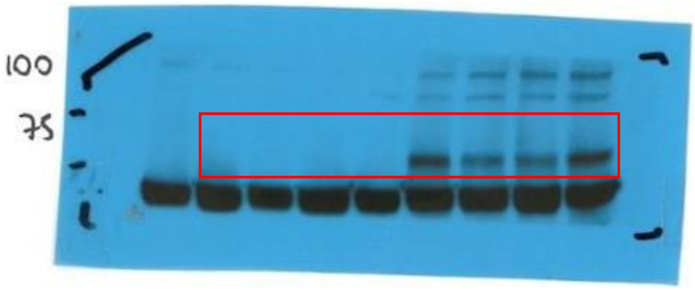

BORA

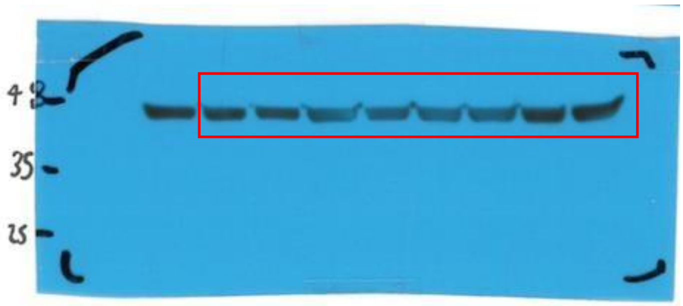

B-Actin

Figure 3A

Figure 3A

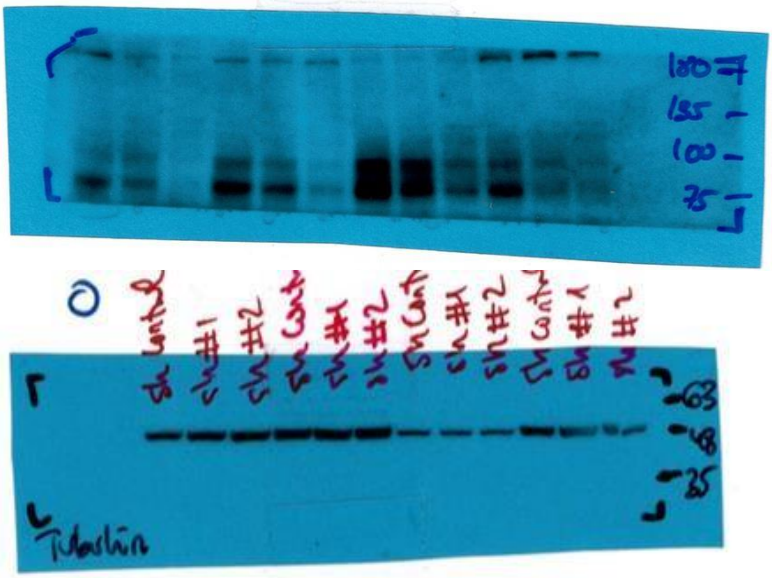

BORA

Tubulin

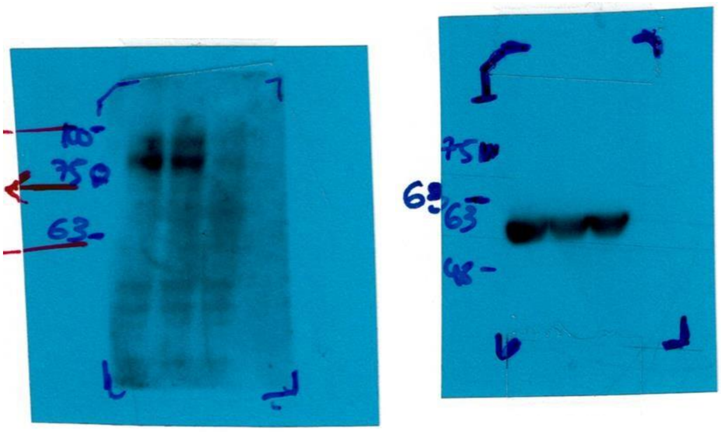

BORA

Figure 3A

Tubulin

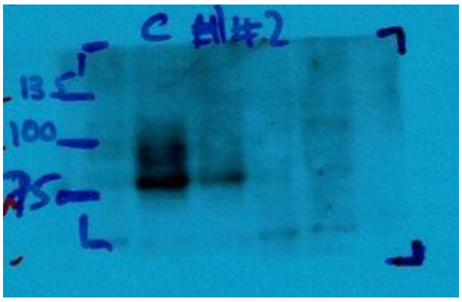

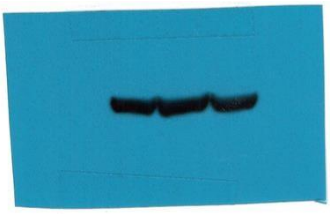

Tubulin

BORA

FIGURE 3E

Figure 3E

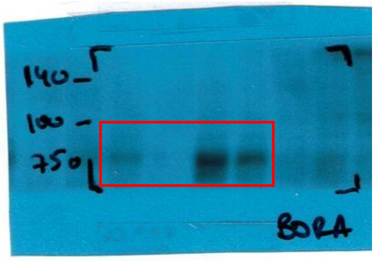

BORA

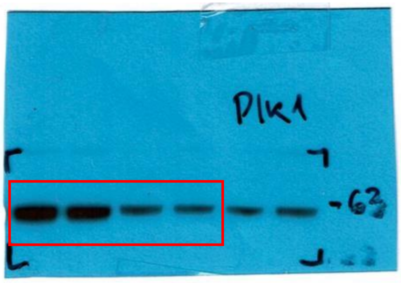

Plk1

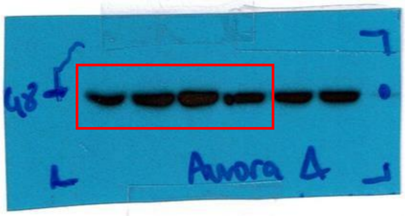

Aurora A

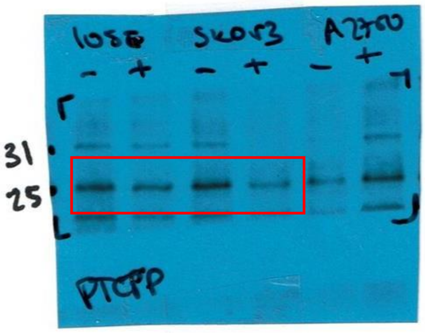

pTCTP (Ser46)

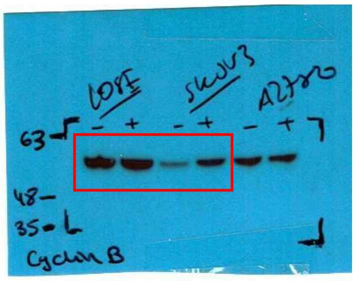

Cyclin B

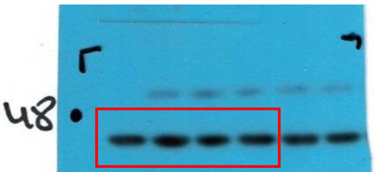

FIGURE 3E

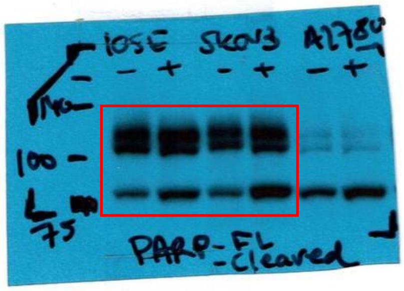

B-Actin

PARP-FL and cleaved

FIGURE 4D

Figure 4D

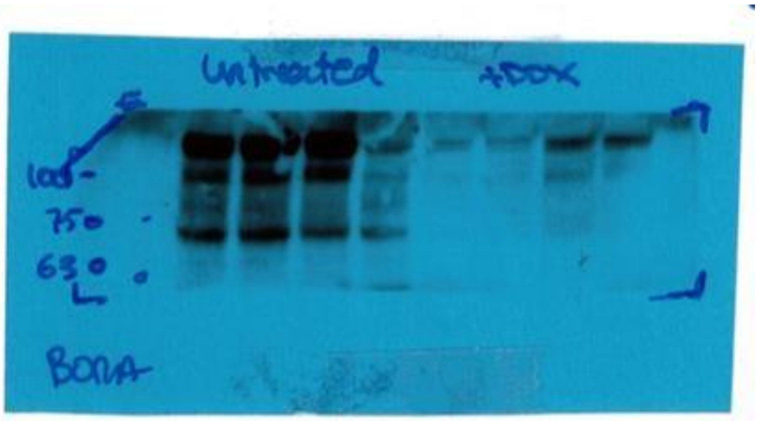

BORA

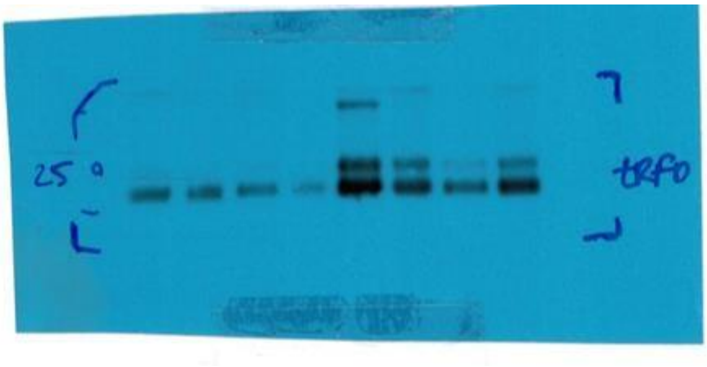

tRFP

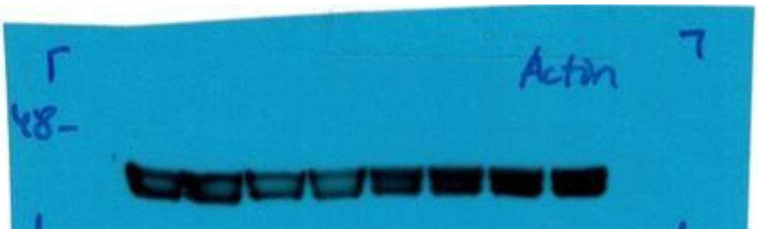

B-Actin

FIGURE 4I

Figure 4I

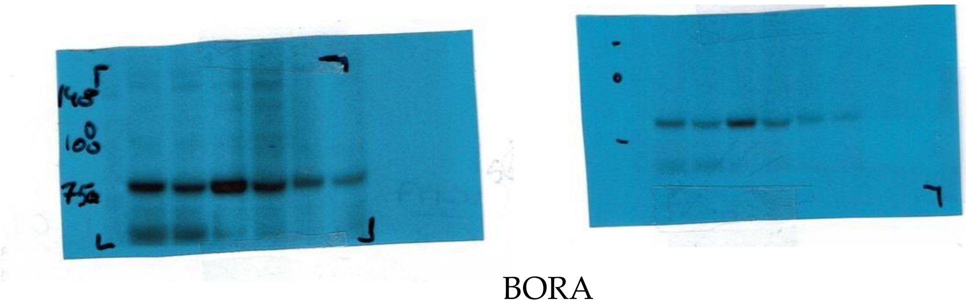

BORA

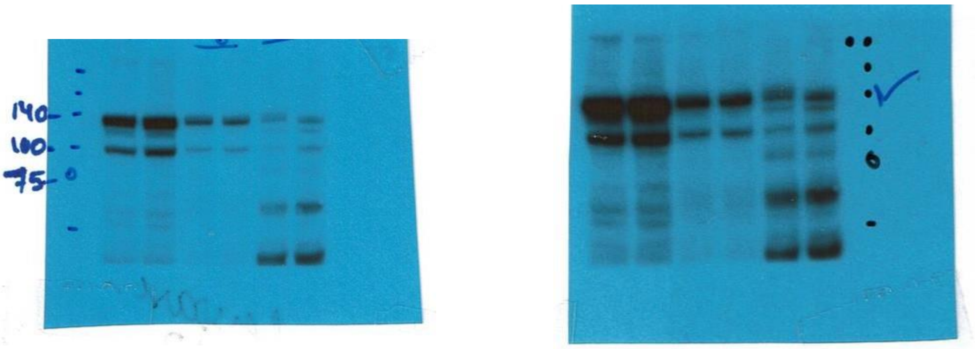

PARP – FL and cleaved

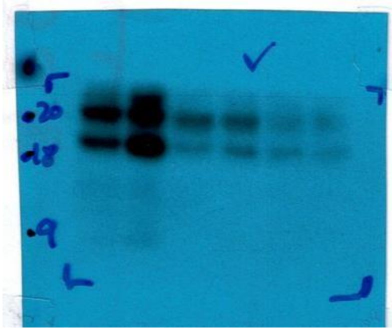

Caspase 3 - cleaved

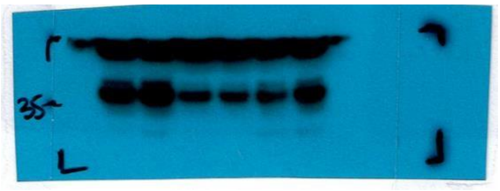

B-Actin

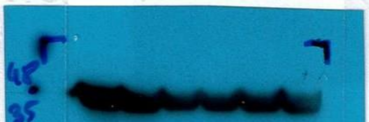

FIGURE 4I

Caspase 3

FIGURE 5H

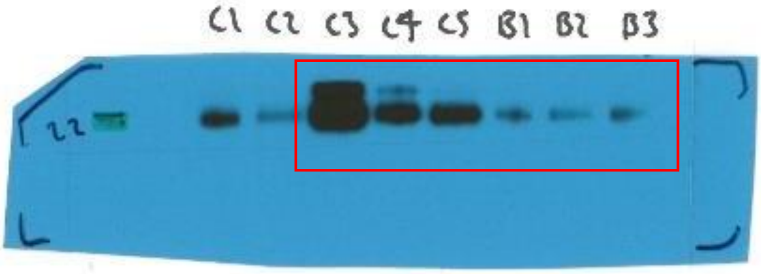

BCL-2

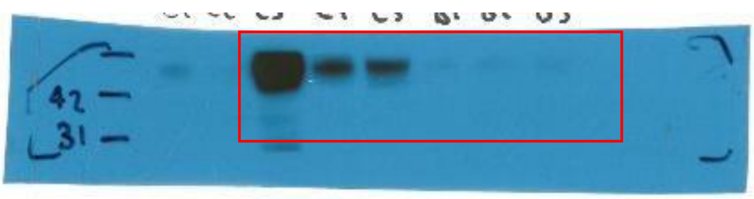

CDK6

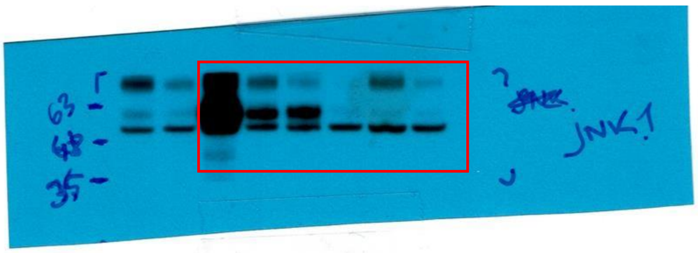

JNK1

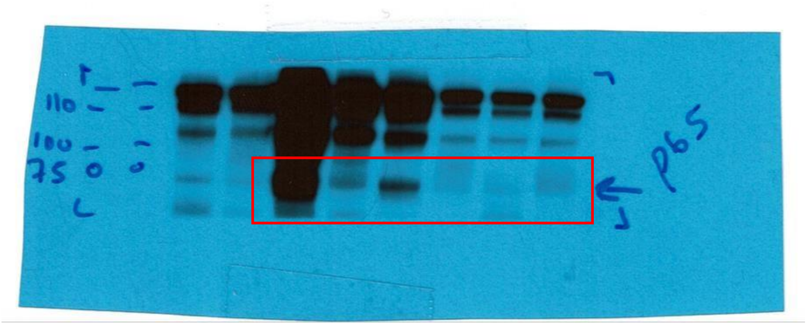

p65

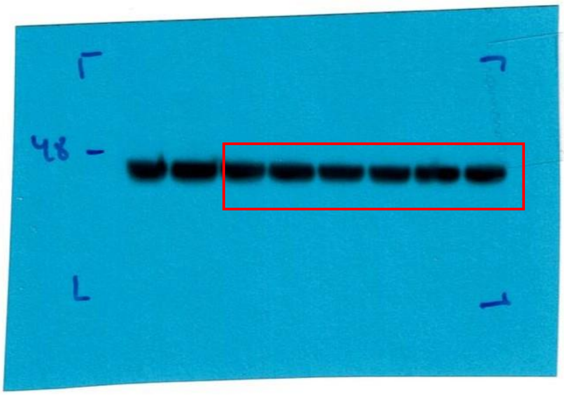

Actin

FIGURE 6E

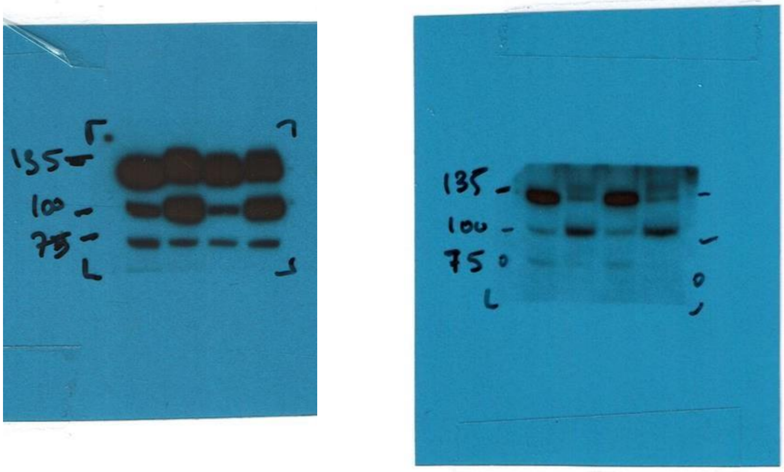

PARP FL y Cleaved

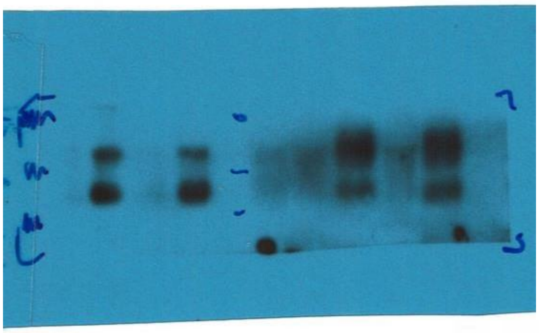

Caspase 3 cleaved

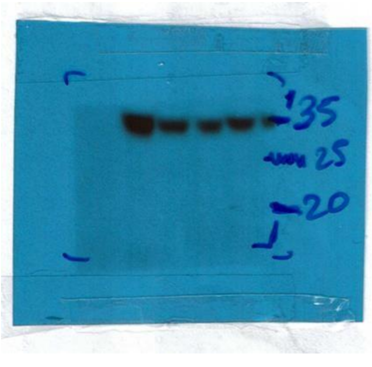

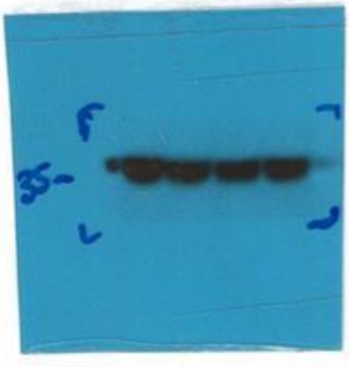

FIGURE 6E

Caspase 3

B-Actin

Supplemental Figure 4a

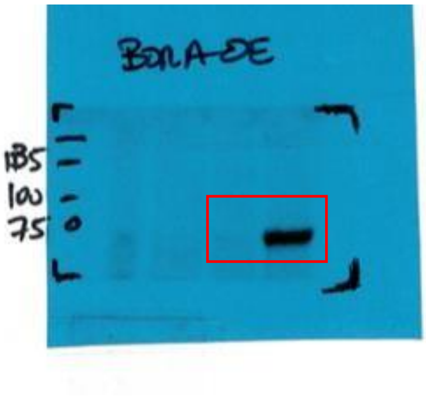

BORA

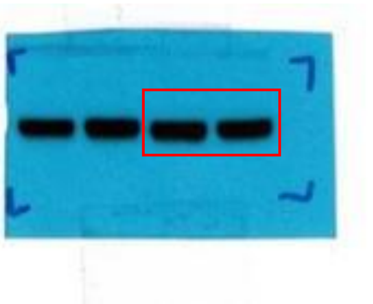

B-Actin

Supplemental Figure 5b

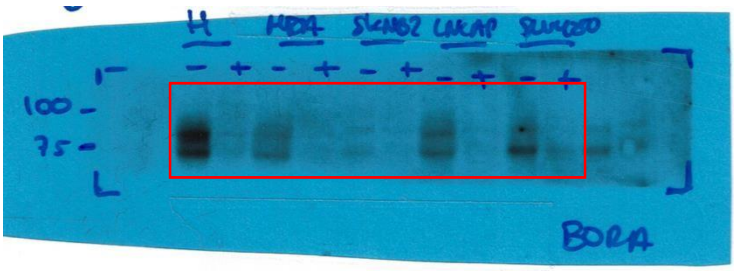

BORA

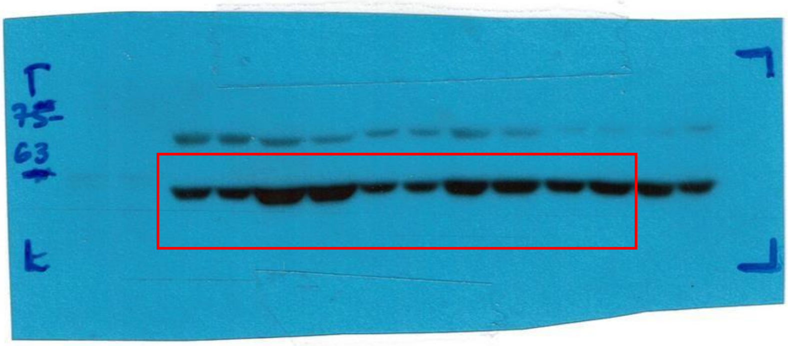

Tubulin

Supplemental Figure 5d

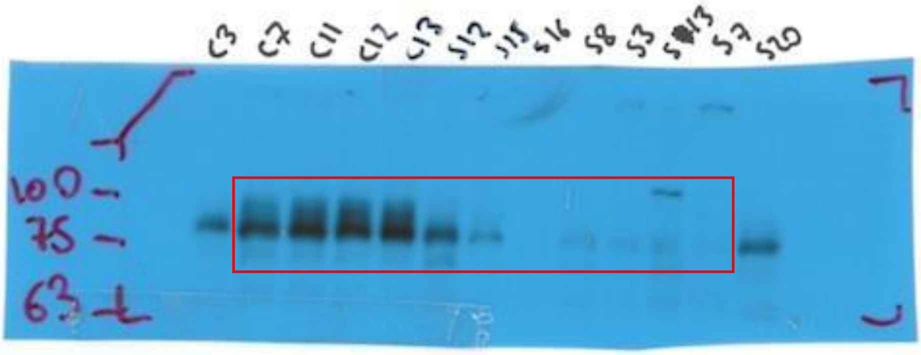

BORA

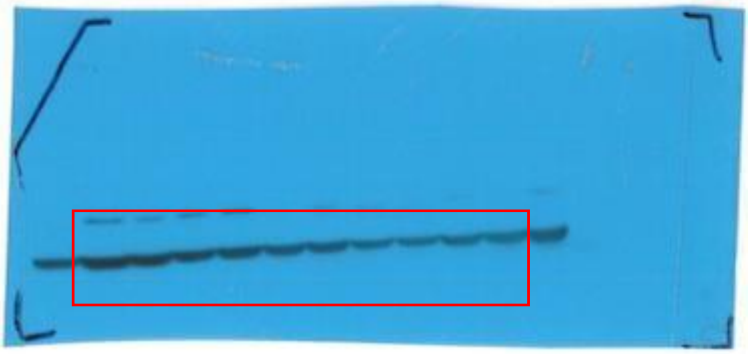

B-Actin

Supplemental Figure 5e

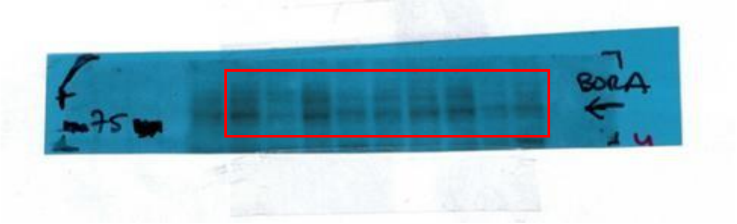

BORA

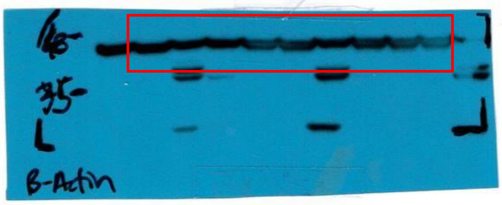

B-Actin

Supplemental Figure 6a

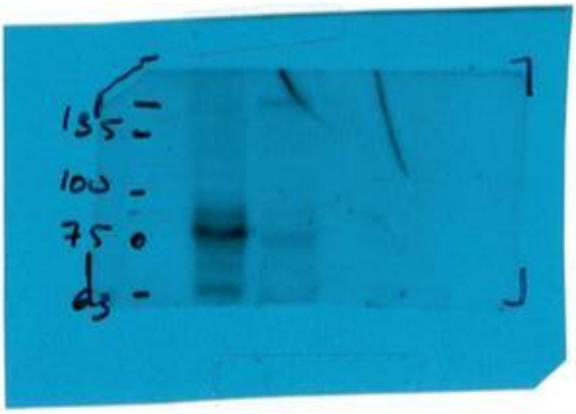

BORA

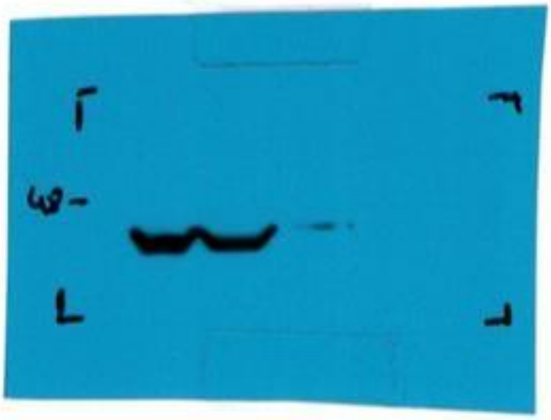

B-Actin

Supplemental Figure 6f

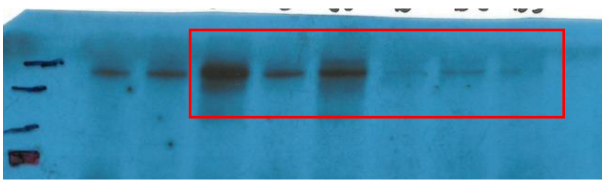

BORA

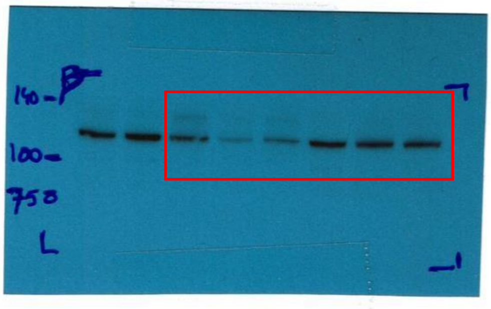

PARP-Full lenght

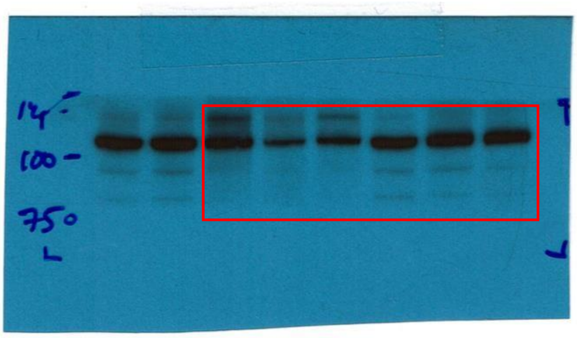

PARP-Cleaved

Supplemental Figure 6f

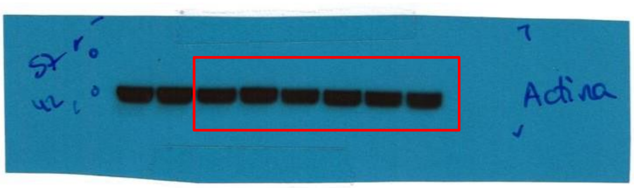

B-Actin

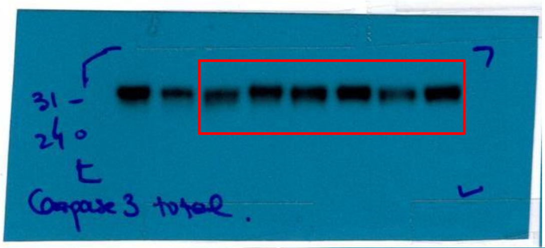

Caspase 3

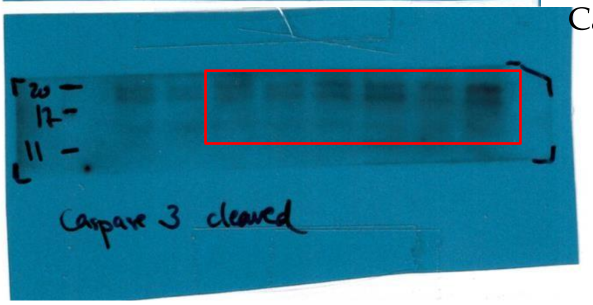

Caspase 3-Cleaved

Supplemental Figure 7b

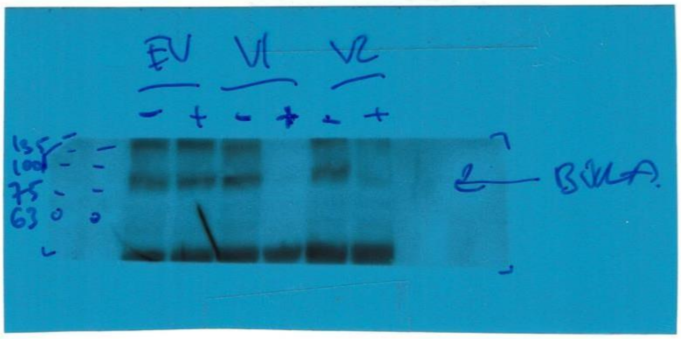

BORA

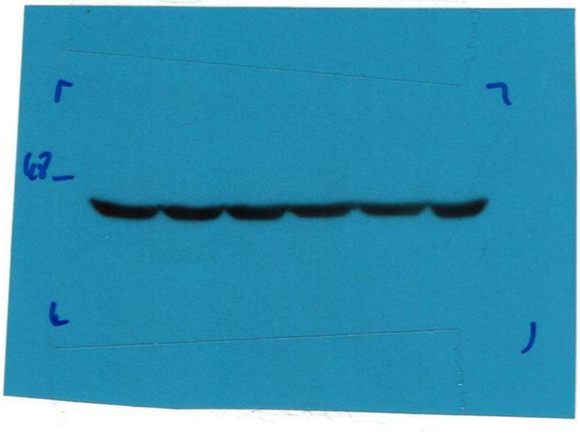

B-Actin

Supplemental Figure 8a

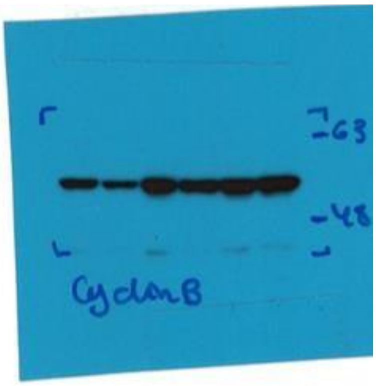

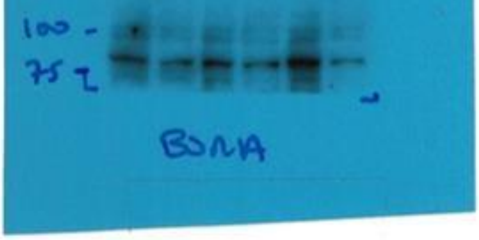

BORA

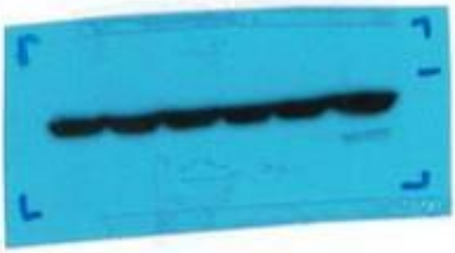

Supplemental Figure 8a

B-Actin

Cyclin B

Supplemental Figure 8a

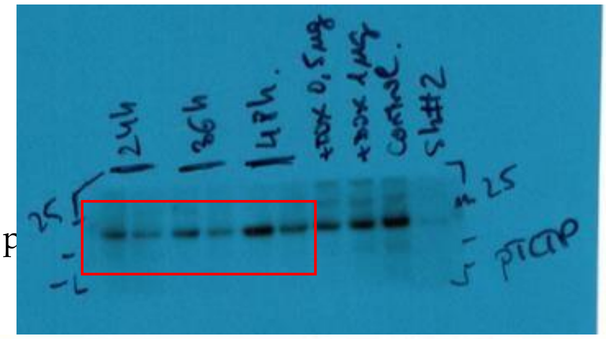

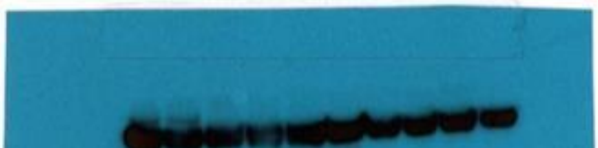

Supplemental Figure 8a

B-Actin
